# Trachealisation of the Oesophagus in Lymphocytic Oesophagitis: An Uncommon Endoscopic Presentation

**DOI:** 10.7759/cureus.98507

**Published:** 2025-12-05

**Authors:** Murshid Ali Mohamed Maheen, Kashif Hameed, Mark R Andrew

**Affiliations:** 1 Gastroenterology, University Hospitals Birmingham NHS Foundation Trust, Birmingham, GBR

**Keywords:** budesonide, dysphagia, eosinophilic oesophagitis, lymphocytic oesophagitis, trachealisation

## Abstract

Lymphocytic oesophagitis (LyE) is a rare histopathological diagnosis with variable and often non-specific endoscopic features. Oesophageal rings and trachealisation are typically associated with eosinophilic oesophagitis but are uncommonly described in LyE. We describe a 79-year-old woman who presented with progressive dysphagia and unintentional weight loss. Endoscopic examination demonstrated trachealisation of the oesophagus, while histopathological evaluation confirmed florid lymphocytic oesophagitis with apoptotic keratinocytes. The patient exhibited only partial symptom relief with proton pump inhibitor (PPI) therapy; however, induction with swallowed budesonide resulted in marked clinical improvement and weight stabilisation. This case highlights an unusual endoscopic manifestation of LyE, expanding the recognised phenotypic spectrum of this entity and emphasising the essential role of histopathological evaluation in distinguishing oesophagitis subtypes.

## Introduction

Lymphocytic oesophagitis (LyE) is a rare subtype of chronic oesophagitis, first described by Rubio et al. in 2006 [1]. It remains an uncommon diagnosis, with population-based studies estimating a prevalence of approximately 0.1-0.2% of all oesophageal biopsies, although higher rates of up to 8-12% have been reported in selected cohorts evaluated for dysphagia [2,3]. It is characterised by a distinct histologic pattern of increased peripapillary intraepithelial lymphocytes with absent or minimal intraepithelial granulocytes. Clinically, LyE presents with variable symptoms, including dysphagia, dyspepsia, nausea, and non-cardiac chest pain [2]. The condition is reported most frequently in middle-aged to older women and has been associated with tobacco use, gastro-oesophageal reflux disease (GERD), and, in some cases, oesophageal motility disorders [2,3].

Histologically, diagnosis depends on identifying peripapillary intraepithelial lymphocytosis, marked intercellular oedema (spongiosis), and minimal or absent eosinophils or neutrophils. Although no universal cut-off exists, a practical threshold of more than 20 intraepithelial lymphocytes per high-power field in the absence of infection is often applied [3]. Endoscopic findings are variable and may range from a normal-appearing oesophagus to mucosal fragility, linear furrows, rings, strictures, white plaques, or oedema. In many cases, the mucosa appears macroscopically normal despite diagnostic histology [2,3]. Because LyE and eosinophilic oesophagitis (EoE) share overlapping clinical symptoms, particularly dysphagia and chest discomfort, and may both exhibit features such as rings, linear furrows, or oedema, the two conditions are frequently confused clinically and endoscopically. However, unlike EoE, LyE lacks a prominent eosinophilic infiltrate, making histology essential for accurate distinction [3,4].

The pathogenesis of LyE remains incompletely understood. Proposed mechanisms include an immune-mediated or hypersensitivity reaction to luminal antigens or a localised autoimmune process [2,3]. It has been recognised as a distinct clinicopathologic entity with features intermediate between reflux-related injury and immune-mediated oesophagitis [1,5]. Recent series have also identified potential risk factors for this condition, including proton pump inhibitors and autoimmune comorbidities, most notably coeliac disease, inflammatory bowel disease (particularly Crohn’s disease), Hashimoto thyroiditis, and, less commonly, systemic lupus erythematosus, although causality remains uncertain [3,6].

## Case presentation

A 79-year-old woman presented with progressive dysphagia to both solids and liquids, associated with unintentional weight loss of approximately one stone over several months. She also reported intermittent dyspepsia, described as episodic upper-abdominal discomfort, and noted that food occasionally felt “stuck” in the lower chest. There was no abdominal pain, odynophagia, or history of atopy. Her appetite had reduced, and she felt increasingly fatigued, although she remained independent in activities of daily living. She was a non-smoker, did not consume alcohol, and had no known autoimmune conditions. Her regular medications included atorvastatin, lansoprazole, ramipril, and sertraline. She reported only partial symptom relief with a 12-week trial of proton pump inhibitor (PPI) therapy.

**Figure 1:**
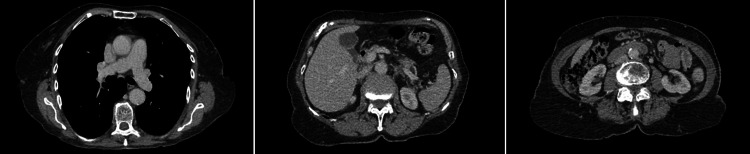
Representative axial CT images of the thorax, abdomen, and pelvis demonstrating no evidence of mediastinal or abdominal lymphadenopathy or mass lesions.

Baseline laboratory investigations revealed no evidence of anaemia or systemic inflammation (Table 1). Computed tomography of the thorax, abdomen, and pelvis (CT TAP) demonstrated no structural abnormalities or masses (Figure 1).

**Table 1:** Summary of routine blood investigations with reference ranges.

Test	Value	Reference Range	Unit
Calcium	2.42	2.20-2.60	mmol/L
Phosphate	1.09	0.80-1.50	mmol/L
Albumin	39	35-50	g/L
Alkaline Phosphatase (ALP)	79	30-130	U/L
Alanine Transaminase (ALT)	15	0-55	U/L
Bilirubin	6	<21	umol/L
Sodium	141	133-146	mmol/L
Urea	6.7	2.5-7.8	mmol/L
Creatinine	74	49-90	umol/L
eGFR	66	>60	mL/min/1.73m^2
White Cell Count	5.71	3.0-10.9	10^9/L
Haemoglobin	115	115-154	g/L
Platelets	254	150-400	10^9/L
C-reactive Protein	3	0-9.9	mg/L
MCV	101.4	81.0-102.0	fL
Ferritin	108	20-235	ng/mL
Vitamin B12	295	187-883	ng/L
Folate	9.9	3.1-20.5	ug/L

**Figure 2:**
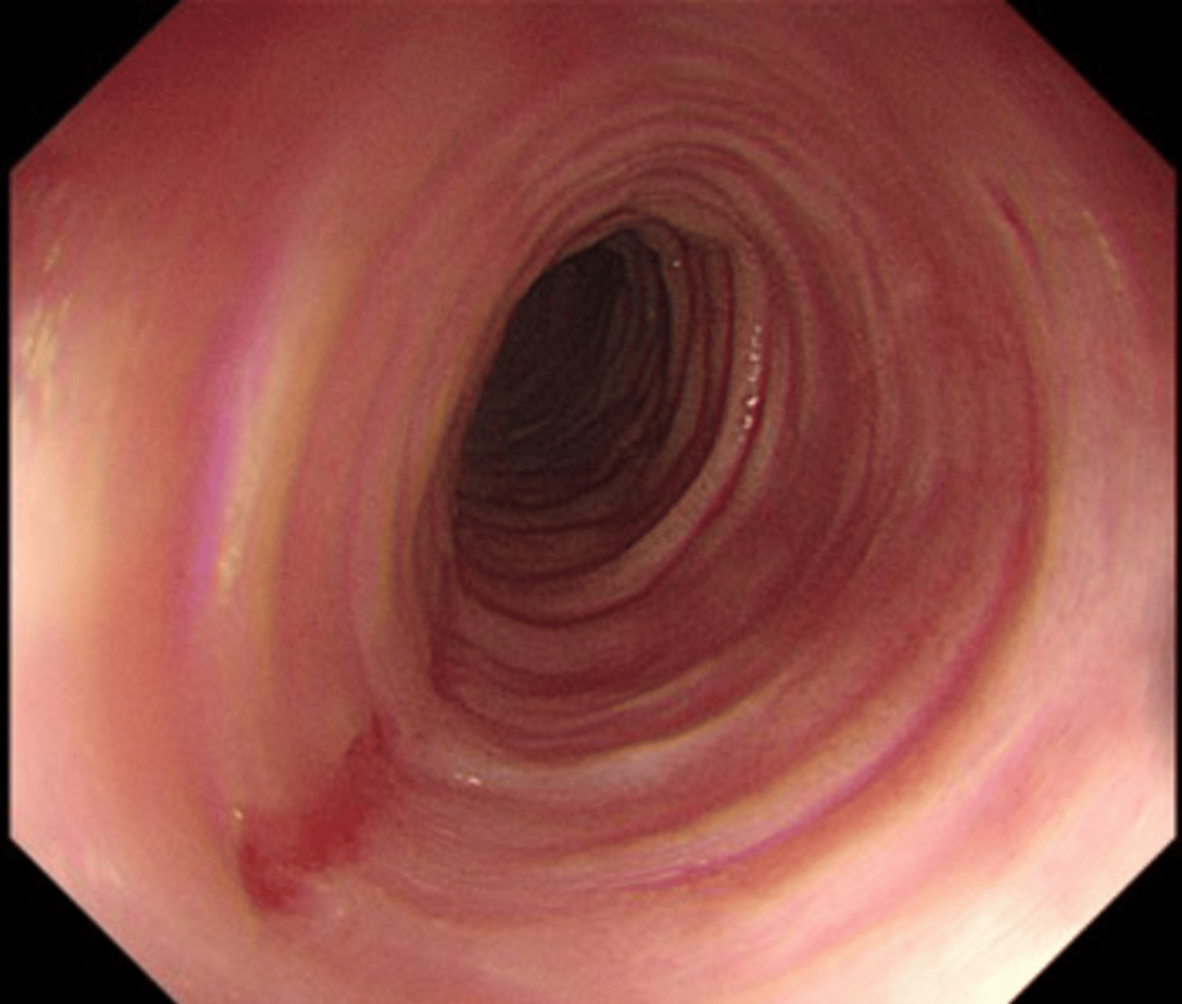
Endoscopic image showing concentric oesophageal rings ('trachealisation') in the mid oesophagus.

**Figure 3:**
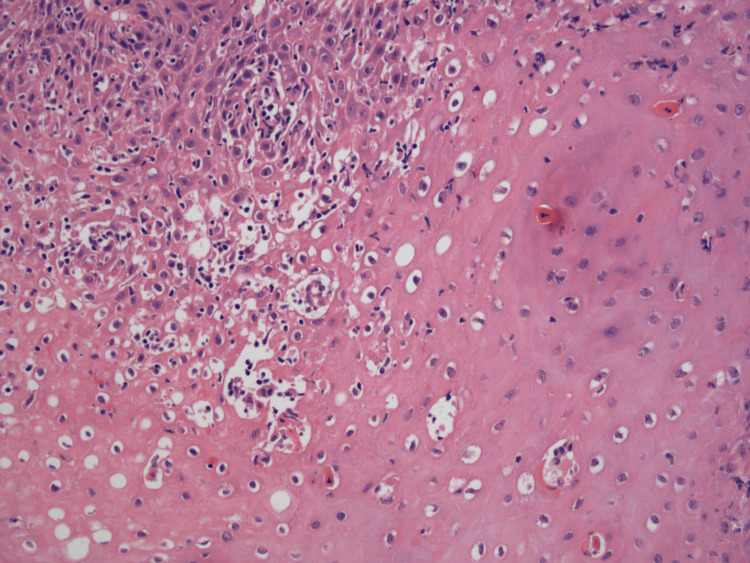
Histopathological section of oesophageal biopsy demonstrating florid lymphocytic oesophagitis with intraepithelial lymphocytosis and apoptotic keratinocytes.

Oesophagogastroduodenoscopy (OGD) revealed multiple concentric rings throughout the oesophagus, giving a characteristic 'trachealisation' appearance (Figure 2). Mucosal biopsies taken from the mid and distal oesophagus demonstrated florid lymphocytic oesophagitis, characterised by a dense intraepithelial lymphocytic infiltrate and scattered apoptotic keratinocytes (Figure 3). Eosinophils and neutrophils were notably absent.

The patient was commenced on a high-dose PPI regimen, with minimal improvement in symptoms. A subsequent trial of swallowed topical budesonide resulted in marked symptomatic relief, with resolution of dysphagia and stabilisation of body weight over follow-up.

## Discussion

LyE is now recognised as a distinct clinicopathologic entity. The hallmark histologic triad consists of dense peripapillary intraepithelial lymphocytic infiltration, intercellular oedema, and the virtual absence of granulocytes [1, 3]. Immunohistochemistry typically reveals T-cell predominance (CD3⁺) with variable CD4⁺ and CD8⁺ expression [4]. Spongiosis is often proportional to the degree of lymphocytic infiltration, reflecting mucosal injury secondary to epithelial immune activation [7]. Clinically, patients most often present with dysphagia or reflux-like symptoms. Because endoscopic findings are frequently minimal or normal, diagnosis relies heavily on histopathological correlation [2,3]. When present, endoscopic abnormalities such as rings, furrows, or strictures suggest chronic inflammatory remodelling and may represent late sequelae of persistent mucosal injury [3,4].

Differentiating LyE from EoE can be challenging, particularly when trachealisation or ringed morphology is observed. Although classically associated with EoE, ring formation has been documented in several reports of LyE, including the case series by Pleet et al. [8], highlighting that concentric oesophageal rings are not pathognomonic for eosinophilic inflammation. In EoE, the histologic hallmark is dense eosinophilic infiltration with associated basal-zone hyperplasia and subepithelial fibrosis [3]. In contrast, our patient’s biopsies demonstrated florid lymphocytic infiltration, apoptotic keratinocytes, and an absence of eosinophils and neutrophils, confirming LyE as the underlying pathology. The occurrence of concentric rings in LyE highlights that ring formation is not pathognomonic of EoE and may occasionally result from chronic lymphocytic inflammation [4,8].

Treatment data for LyE are limited. Unlike EoE, where food-triggered antigenic responses are central to pathogenesis, there is currently no evidence to support the use of elimination diets in LyE, and published series have not demonstrated symptomatic or histological benefit with dietary restriction [3,5]. Empirical high-dose proton pump inhibitor (PPI) therapy is often trialled first, though the response is variable [2,3]. Swallowed topical corticosteroids such as budesonide or fluticasone have been reported to achieve both clinical and histologic improvement in small series [3]. In our case, the patient achieved complete symptom resolution and weight stabilisation following topical budesonide therapy, supporting a steroid-responsive inflammatory mechanism.

## Conclusions

This case highlights an unusual endoscopic manifestation of trachealisation (concentric oesophageal rings) in lymphocytic oesophagitis, a finding more commonly associated with eosinophilic oesophagitis. It emphasises that ringed oesophageal morphology should not automatically be attributed to EoE and that histological confirmation remains essential for accurate diagnosis. The favourable clinical response to budesonide in this elderly patient further underscores the therapeutic potential of topical corticosteroids in LyE. Recognition of this distinct but underdiagnosed entity is important, as early identification and appropriate treatment can lead to significant symptomatic improvement and prevent chronic remodelling of the oesophageal mucosa. A limitation of this report is the lack of follow-up histology and the inherent challenges in fully excluding competing causes of oesophageal injury in a single-patient case, which should be considered when interpreting the findings. Further research is needed to clarify the underlying pathophysiology of LyE, establish evidence-based treatment strategies, and better define its natural history and optimal management.
